# No evidence for an association between in utero Ramadan exposure and mean arterial pressure and random blood glucose in adulthood: evidence from SEACO in Malaysia

**DOI:** 10.1017/jns.2025.10060

**Published:** 2025-12-05

**Authors:** Patricia Mary Elizabeth, Fabienne Pradella, Tin Tin Su, Andrea U. Seiermann, Anja Schoeps, Roshidi Ismail, Reyn van Ewijk, Volker Winkler, Melani R. Mahanani

**Affiliations:** 1 Heidelberg Institute of Global Health, Heidelberg University Hospital, Heidelberg, Germany; 2 Statistics and Econometrics, Faculty of Law, Management and Economics, Johannes Gutenberg-University, Mainz, Germany; 3 Division of Primary Care and Population Health, Department of Medicine, Stanford University, Stanford, CA, USA; 4 South East Asia Community Observatory (SEACO), Jeffrey Cheah School of Medicine and Health Sciences, Monash University Malaysia, Bandar Sunway, Malaysia; 5 Department of Anaesthesiology, Hospital Center Biel/Bienne, Biel/Bienn, Switzerland; 6 Centre for Preventive Medicine and Digital Health (CPD), Division of Prevention of Cardiovascular and Metabolic Diseases, Medical Faculty Mannheim, Heidelberg University, Mannheim, Germany

**Keywords:** foetal programming, In utero Ramadan, mean arterial pressure, random blood glucose, pregnancy, MAP, Mean arterial pressure, RBG, Random blood glucose, SEACO HDSS, Southeast Asia community observatory health and demographic surveillance system, DiD, Difference-in-differences

## Abstract

A growing body of evidence shows an association between in utero Ramadan exposure and negative long-term consequences. Nonetheless, there is a scarcity of studies utilizing clinical measures in adults. This study investigates a possible association between in utero Ramadan exposure and mean arterial pressure (MAP) as well as random blood glucose (RBG) measures in the adult offspring. Using cross-sectional data from the Southeast Asia community observatory health and demographic surveillance system (SEACO) in Malaysia for two survey rounds (year 2013 and 2018), we compared MAP and RBG of in utero Ramadan-exposed Muslims with unexposed Muslims and non-Muslims. In utero Ramadan exposure was estimated based on the overlap between pregnancy (estimated from birth dates) and Ramadan periods. We conducted difference-in-differences analyses adjusted for age and birth months (seasonal effects). A total of 20,575 participants aged 35 or older were included in the analysis, comprising 12,696 Muslims and 7,879 non-Muslims. Difference-in-differences analyses revealed no statistically significant association between in utero Ramadan exposure and MAP, or between in utero Ramadan and RBG. These findings persisted in additional analyses examining the timing of Ramadan exposure during pregnancy.

## Introduction

Ramadan is one of the five pillars of Islam, during which most Muslims are obliged to abstain from eating, drinking, smoking, sexual intercourse, and taking oral medicine from dawn to sunset for about 30 days. The timing of meals, the type of food consumed, sleep, and other behavioural patterns are also changed.^(1)^ However, certain groups of people may refrain from Ramadan fasting, including children before the age of puberty, the sick, the elderly, travellers without the option of fasting, women in their menstrual period, women in the post-partum, breastfeeding women, and pregnant women.^(2,3)^ These groups should make up for the fasting days they missed by either fasting on their own at a later time or by making food or monetary donations (known as Fidya).^(4)^ Although pregnant women are exempt from fasting during Ramadan, surveys conducted in predominantly Muslim countries, such as Malaysia, Iraq, Iran, Pakistan, Indonesia, and Bangladesh, indicate that the majority of expecting Muslim mothers (approximately 60%–99%) still choose to fast for social or religious reasons.^(3,5–11)^ The survey conducted in Malaysia also found no significant differences in their understanding of Islamic law between fasting and non-fasting groups.^(5)^

Physiologically, fasting induces hypoglycaemia, a condition in which the blood sugar level is lower than the standard range. As one compensatory mechanism, the human body increases fat metabolism, which produces ketones as a byproduct. High concentrations of ketones can lower the blood pH to dangerous levels.^(12)^ Due to the increased energy demand for the placenta and foetus, pregnant women may develop hypoglycaemia and ketoacidosis faster, especially during the day when fasting coincides with increased physical activities.^(13)^ The theory of foetal programming describes the phenomenon in which alteration of maternal nutrition during pregnancy may result in changes in foetal genome expression, which in turn, can lead to negative long-term health outcomes.^(14)^

In healthy non-pregnant adults, Ramadan fasting is associated with lowered blood pressures, fat mass and body mass regardless calorie intake changes, improved lipid profile, attenuation of systemic low-grade inflammation as well as transient alterations of gut microbiota independent of living area and diet composition.^(15–18)^ Meanwhile, there is evidence that in utero exposure to Ramadan adversely affects the health offspring, both from systematic review focusing on long-term effects^(19)^ and from systematic review and meta-analysis conducted on both short- and long-term effects,^(20)^ which have reported the associations with increased rates of under-five mortality,^(21)^ shorter stature,^(22)^ lower body mass index,^(22,23)^ poorer cognitive performance, and higher incidence of vision, hearing, and learning disabilities.^(24)^ Another study reported an increased risk for symptoms indicative of chronic diseases such as coronary heart disease and type 2 diabetes mellitus.^(2)^ As the majority of studies investigate self-reported diagnoses of health conditions, there is a scarcity of studies utilizing clinical measures of risk factors for chronic conditions in adult offspring. To the best of our knowledge, similar studies have been conducted using pulse pressure, blood pressure, and fasting blood insulin.^(2,25)^ Our study aimed to investigate the association between in utero Ramadan exposure and mean arterial pressure (MAP) and random blood glucose (RBG) measures in adult offspring.

## Methods

### Data

In Malaysia, Islam is the federal religion and religious practices are an integral part of daily life.^(26)^ In Segamat, Malaysia, the Southeast Asia Community Observatory health and demographic surveillance system (SEACO HDSS) conducted a household-level health survey for the first time in 2013. The SEACO data collection team conducted annual visits to enumerate and update information on all individuals living in five of 11 sub-districts in Segamat, namely Bekok, Chaah, Gemereh, Jabi, and Sungai Segamat. Each household was visited up to three times if no one was present during the first two attempts. Since all individuals in the SEACO sub-districts had an equal opportunity to participate, selection bias was minimized. The collected data provide information on the general health of half of the estimated total population, including medical history, psychological well-being, risk factors for non-communicable disease and use of health services.^(27)^

The data comprised all observations from the two combined SEACO HDSS survey rounds in 2013 and 2018. Twenty-five percent of the individuals participated in both surveys; thus, only their second observation was included in the analysis. Further, only participants aged 35 years or above with plausible reported age (age between the date of interview and the date of birth) were included as blood pressure and blood glucose measures were not available for younger individuals. The final dataset included information on sex, date of birth, date of interview, age at interview, religion, and measures of blood pressure and RBG.

### Outcome measures

The SEACO HDSS data collection teams employed a digital automatic blood pressure device to measure the blood pressure of each participant on their left arm on three occasions, with a three-minute interval between each measurement. Prior to the initial blood pressure measurement, the participants were required to remain seated for a minimum of 15 minutes.^(28)^ MAP was calculated using the second and third measurements, with one-third of the value attributed to the systolic and two-thirds attributed to the diastolic blood pressure.^(29)^ RBG was determined from blood samples collected without prior fasting.^(27)^

### Ramadan exposure categories

Following the literature, we estimated in utero Ramadan exposure based on date of birth. Specifically, we calculated the overlap between date of birth and the historical Ramadan period in Malaysia, assuming an average pregnancy duration of 266 days after the conception.^(2,30)^ The exposure was categorized to the following seven groups: (i) ‘certainly not exposed’, (ii) ‘probably not exposed’, (iii) ‘conceived during Ramadan’, (iv) ‘Ramadan during the first trimester’, (v) ‘Ramadan during the second trimester’, (vi) ‘Ramadan during the third trimester’, (vii) ‘born during Ramadan’ (see Table 1).

**Table 1.** Categorization of estimated in utero Ramadan exposure for different analysis

Group	Name	Description
(i)	Certainly not exposed	No Ramadan between 20 days before conception and date of birth
(ii)	Probably not exposed	Ramadan between 20 days before conception and conception, but no Ramadan between conception and date of birth
(iii)	Conceived during Ramadan	Conception happened during Ramadan
(iv)	Ramadan during the 1^st^ trimester	Ramadan started during the first 84 days after conception
(v)	Ramadan during the 2^nd^ trimester	Ramadan started between day 85 and day 182 of pregnancy
(vi)	Ramadan during the 3^rd^ trimester	Ramadan started between a day after the second trimester and until a day before the date of birth
(vii)	Born during Ramadan	Date of birth was in Ramadan month

### Statistical analyses

First, we checked for birth heaping using a bar graph and tested for pregnancy planning avoiding Ramadan by comparing the observed number of conceptions during Ramadan with the expected number assuming an even distribution using a two-sided binomial probability test.

Difference-in-differences (DiD) analyses were conducted using ‘being Muslim’, ‘being in utero during Ramadan (in different phases of gestation)’, and the interaction term ‘being Muslim 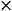 being in utero during Ramadan’ utilizing different exposure categories as independent variables. Since non-Muslims do not observe Ramadan fasting, they act as a control group for residual confounding in the comparison between being in utero during Ramadan and not being in utero during Ramadan.

DiD Model 1 looked at a binary exposure by combining the exposure categories mentioned above into exposed (group (iii) to group (vii)) and not exposed (group (i) and group (ii)). Model 2 utilized again group (iii) to group (vii) as the exposure category but the unexposed are considered separately using group (i) and group (ii). Finally, Model 3 considered the timing of Ramadan exposure during pregnancy by using all exposure groups as independent variables. All models were separated by sex and adjusted for age, age squared, and month of birth to control for birth’s seasonal effect on lifetime disease risks.^(31–33)^

In a final step, we performed different robustness checks. First, we fitted logistic regression models to evaluate the association between Ramadan exposure and the likelihood of receiving treatment for hypertension or hyperglycaemia as well as a full set of interaction terms of Ramadan exposure with Muslim and the original controls. Second, to reflect the greater burden of hypertension and hyperglycaemia in later life, we re-estimated our primary models in the subsample aged ≥50 years, where high blood pressure and high blood glucose are more prevalent.^(34,35)^

All statistical analyses were done using Stata version 17.0 (StataCorp LLC, 4905 Lakeway Drive, College Station, TX 77845, USA).

## Results

Descriptive characteristics stratified by religion are depicted in Table 2. Blood pressure and RBG measures were collected from a total of 20,575 people, consisting of 12,696 Muslims and 7,879 non-Muslims. On average, Muslims were younger and had higher systolic and diastolic blood pressures than non-Muslims, while there was no difference with respect to RBG.

**Table 2.** Characteristics of 12,696 Muslims and 7,879 Non-Muslims in Malaysia (Age ≥35 Years)

		Religion				
**Characteristics**	**Non-Muslim**			**Muslim**		
	***N***	**%**	**SD**	***N***	**%**	**SD**
Total	7,879			12,696		
Mean age (years)	58.5		12.8	56.0		12.5
5-year age group						
35–39	635	8.1%		1,431	11.3%	
40–44	621	7.9%		1,383	10.9%	
45–49	821	10.4%		1,453	11.4%	
50–54	1,001	12.7%		1,571	12.4%	
55–59	1,123	14.2%		1,727	13.6%	
60–64	1,117	14.2%		1,830	14.4%	
65–69	923	11.7%		1,443	11.4%	
≥70	1,638	20.8%		1,858	14.6%	
Sex						
Male	3,507	44.5%		5,747	45.3%	
Female	4,372	55.5%		6,949	54.7%	
Birth months						
January	631	8.0%		1,111	8.7%	
February	583	7.4%		900	7.1%	
March	682	8.7%		1,046	8.2%	
April	625	7.9%		1,114	8.8%	
May	703	8.9%		1,179	9.3%	
June	635	8.1%		1,077	8.5%	
July	686	8.7%		1,176	9.3%	
August	696	8.9%		1,019	8.0%	
September	624	7.9%		1,020	8.0%	
October	688	8.7%		1,055	8.3%	
November	710	9.0%		998	7.9%	
December	616	7.8%		1,001	7.9%	
Ramadan exposure category						
Certainly not in utero during Ramadan	811	10.3%		1,291	10.2%	
Probably not in utero during Ramadan	439	5.6%		703	5.5%	
In utero during Ramadan	6,629	84.1%		10,702	84.3%	
Conceived during Ramadan	679	10.2%		1,075	10.0%	
Ramadan started in trimester 1	1,961	29.6%		3,069	28.8%	
Ramadan started in trimester 2	2,158	32.6%		3,513	32.8%	
Ramadan started in trimester 3	1,210	18.2%		2,017	18.8%	
Born during Ramadan	621	9.4%		1,028	9.6%	
Mean systolic blood pressure (mmHg)	131.1		18.9	133.8		20.3
Mean diastolic blood pressure (mmHg)	76.5		11.2	78.6		11.2
Mean arterial pressure (mmHg)	94.7		12.3	97.0		12.8
Mean random blood glucose (mmol/L)	8.0		3.6	8.1		3.9

The bar chart did not reveal strong birth heaping effects (see Appendix Figure A1). In addition, no significant difference between expected and observed number of conceptions during Ramadan from a two-sided chi-squared test was observed (p-value 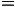 0.240). Tables 3 and 4 present the result of the DiD analyses. Both analyses showed no significant associations between being in utero during Ramadan and differences in MAP or RBG, respectively. The robustness checks using logistics regressions with medication use as outcome resulted in no difference in the likelihood of antihypertensive or antihyperglycaemic medication (see Appendix Table A1). Further, restricting the analysis to those aged ≥50 years with identical model specification yielded effect estimates that remained statistically non-significant and of similar magnitude to those obtained for the full sample (Appendix Tables A2–A3).

**Table 3.** Association between in utero Ramadan exposure and mean arterial pressure in adult offspring (≥35 years old, *n* = 20,528), by sex (only interaction terms are shown)

Variables	Female		Male	
	Coef.	p-value	Coef.	p-value
Model 1* (adj. R^2^ 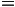 0.03 and 0.01)				
Muslim × Not exposed (group (i) + (ii))	Ref.	Ref.	Ref.	Ref.
Muslim × In utero Ramadan (group (iii) to (vii))	−0.66	0.33	−0.26	0.72
Model 2^†^ (not exposed vs. exposed/probably not exposed) (adj. R^2^ 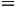 0.03 and 0.01)				
Muslim × Certainly not exposed (group (i))	Ref.	Ref.	Ref.	Ref.
Muslim × Probably not exposed (group (ii))	0.99	0.44	−2.01	0.15
Muslim × In utero Ramadan (group (iii) to (vii)	−0.30	0.71	−0.95	0.27
Model 3^‡^ (adj. R^2^ 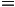 0.03 and 0.02)				
Muslim × Certainly not exposed (group (i))	Ref.	Ref.	Ref.	Ref.
Muslim × Probably not exposed (group (ii))	0.99	0.44	−2.00	0.15
Muslim × Conceived during Ramadan (group (iii))	−1.17	0.30	0.01	0.99
Muslim × Ramadan during the first trimester (group (iv))	−0.36	0.69	−1.62	0.10
Muslim × Ramadan during the second trimester (group (v))	−0.58	0.52	−0.61	0.53
Muslim × Ramadan during the third trimester (group (vi))	0.57	0.57	−1.38	0.19
Muslim × Born during Ramadan (group (vii))	0.10	0.93	−0.10	0.93

*Note:*

* Adjusted for age, age^2^, birth months (categorical), Muslim, not exposed, exposed.

† Adjusted for age, age^2^, birth months (categorical), Muslim, probably not exposed, exposed.

‡ Adjusted for age, age^2^, birth months (categorical), Muslim, probably not exposed, conceived during Ramadan, Ramadan during the first trimester, Ramadan during the second trimester, Ramadan during the third trimester, and born during Ramadan.

**Table 4.** Association between in utero Ramadan exposure and random blood glucose in adult offspring (≥35 years old, *n* = 20,528), by sex (only interaction terms are shown)

Variables	Female		Male	
	Coef.	p-value	Coef.	p-value
Model 1* (adj. R^2^ 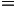 0.03 and 0.02)				
Muslim × Not exposed (group (i) + (ii))	Ref.	Ref.	Ref.	Ref.
Muslim × In utero Ramadan (group (iii) to (vii))	−0.22	0.28	−0.05	0.83
Model 2^†^ (not exposed vs. exposed/probably not exposed) (adj. R^2^ 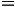 0.03 and 0.02)				
Muslim × Certainly not exposed (group (i))	Ref.	Ref.	Ref.	Ref.
Muslim × Probably not exposed (group (ii))	0.07	0.86	−0.33	0.43
Muslim × In utero Ramadan (group (iii) to (vii))	−0.19	0.44	−0.16	0.54
Model 3^‡^ (adj. R^2^ 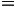 0.03 and 0.02)				
Muslim × Certainly not exposed (group (i))	Ref.	Ref.	Ref.	Ref.
Muslim × Probably not exposed (group (ii))	0.07	0.86	−0.33	0.43
Muslim × Conceived during Ramadan (group (iii))	−0.24	0.49	0.16	0.66
Muslim × Ramadan during the first trimester (group (iv))	−0.09	0.76	−0.15	0.60
Muslim × Ramadan during the second trimester (group (v))	−0.31	0.26	−0.20	0.48
Muslim × Ramadan during the third trimester (group (vi))	−0.18	0.54	−0.11	0.74
Muslim × Born during Ramadan (group (vii))	−0.11	0.76	−0.47	0.21

*Note:*

* Adjusted for age, age^2^, birth months (categorical), Muslim, not exposed, exposed.

† Adjusted for age, age^2^, birth months (categorical), Muslim, probably not exposed, exposed.

‡ Adjusted for age, age^2^, birth months (categorical), Muslim, probably not exposed, conceived during Ramadan, Ramadan during the first trimester, Ramadan during the second trimester, Ramadan during the third trimester, and born during Ramadan.

## Discussion

In this study, we investigated the potential association between in utero Ramadan exposure and the risk of chronic conditions in adult offspring by examining clinical measures of MAP and RBG. Utilizing difference-in-differences analyses, we found no significant association between in utero Ramadan exposure and either MAP or RBG. These findings remained consistent across various analyses, including those considering the timing of Ramadan exposure during pregnancy and multiple robustness checks. Our findings for MAP are in line with another study from Indonesia and even with a famine study utilizing blood pressure,^(2,36)^ however in contrast to findings with higher pulse pressure among the exposed in the Indonesian in utero Ramadan study.^(2)^ Meanwhile, our study was the first one looking at RBG to investigate the association of in utero Ramadan exposure with blood glucose level.

The rationale for analysing MAP rather than systolic or diastolic blood pressures was twofold. Firstly, it has been established that MAP enhances the precision of hypertension diagnosis.^(29,37,38)^ Secondly, it has been observed that there are disparate tendencies in the increase of systolic and diastolic blood pressures with age.^(29,38)^ Studies have shown that blood pressure of the adult offspring was positively associated with the carbohydrate/protein ratio and not associated with total caloric, protein, carbohydrate, or fat intake during pregnancy.^(39,40)^

There are two main study limitations that need to be discussed in more detail before drawing conclusions on a possible association between being exposed to in utero Ramadan and long-term outcomes on MAP and RBG. The first limitation is related to our exposure, which is defined as the overlap between the time in utero and a Ramadan. This intention-to-treat set-up is standard in the literature on Ramadan during pregnancy.^(2,20)^ However, it also implies that we may underestimate the true effect because also children of non-fasting mothers were classified as exposed, if their time in utero overlapped with a Ramadan. Since exposure was calculated based on the date of birth, misreporting of birth dates could have introduced bias toward zero. Furthermore, to reduce exposure misclassification in the absence of gestational age information, we categorized children estimated to have been conceived less than 21 days after the end of Ramadan as ‘probably not exposed’. If these pregnancies were longer than average, their classification as unexposed would be incorrect. Conversely, preterm births can only result in erroneous classification as exposed, introducing additional potential bias toward the null. Even though thus unlikely, severe misclassification might explain smaller non-significant effects but would less likely remove the association completely. Second, RBG, one of the possible diagnostic parameters adopted in the Malaysian guidelines,^(41)^ is less sensitive than fasting blood glucose test or HbA1c which might lead to underestimate a possible effect.^(42,43)^ However, data regarding fasting blood glucose and HbA1c were not available. Nonetheless, our robustness check showed that medication use did not differ by exposure status. This indicates that any pharmacologically induced normalization of RBG or blood pressure is unlikely to have biased our primary estimates.

In conclusion, an absence of an association was observed between in utero Ramadan exposure and MAP or RBG. In view of the aforementioned limitations, we recommend that further research should be conducted using the same clinical measures to replicate and validate our findings. In particular, studies utilizing dietary records to assess general dietary habits and those specifically related to Ramadan would greatly enhance the interpretation of the findings because alterations in macro- and micro-nutrient intake during Ramadan can be varied across different countries and generates a divergence in in utero Ramadan’s effects. Furthermore, alterations in stress levels and sleep patterns during Ramadan should be taken into account, as Ramadan may influence dietary patterns in addition to other factors.^(44)^

## Supporting information

Elizabeth et al. supplementary material Elizabeth et al. supplementary material

## Data Availability

The data supporting the findings of this study are available from the SEACO. Access to the data can be requested through SEACO, subject to relevant data-sharing agreements. Interested parties can contact SEACO for more information on the application process and any conditions associated with the data use.

## Supplementary material

The supplementary material for this article can be found at https://doi.org/10.1017/jns.2025.10060
