# Supplementary material for: No evidence for an association between in utero Ramadan exposure and mean arterial pressure and random blood glucose in adulthood: evidence from SEACO in Malaysia
Source: J Nutr Sci. 2025 Dec 5;14:e87. doi: 10.1017/jns.2025.10060 (PMC12722045; doi:10.1017/jns.2025.10060)
Supplement: Elizabeth et al. supplementary material [file S2048679025100608sup001.docx]

**Figure A1**: Bar graph of birth months for 20,575 participants aged ≥35 years


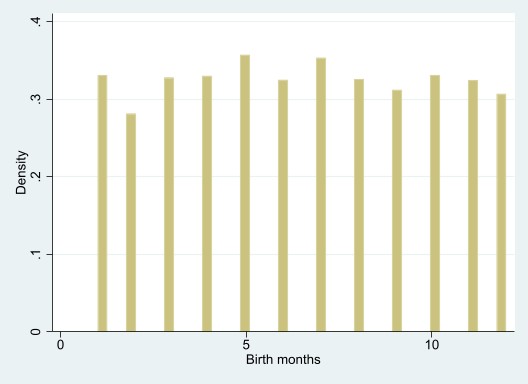


**Table A1.** Robustness checks for the association between in utero Ramadan exposure and medication use

| **Variables** | **Antihypertensive medication use (OR, 95% CI)** | **Antihyperglycemic medication use (OR, 95% CI)** |
| --- | --- | --- |
| Interaction between religion and Ramadan exposure |  |  |
| Muslim x Certainly not exposed | Ref. | Ref. |
| Muslim x Probably not exposed | 0.99 (0.66-1.50) | 2.07 (0.93-4.62) |
| Muslim x Conceived during Ramadan | 0.73 (0.50-1.06) | 1.48 (0.76-2.90) |
| Muslim x Ramadan during the first trimester | 0.87 (0.65-1.16) | 1.03 (0.61-1.74) |
| Muslim x Ramadan during the second trimester | 0.99 (0.74-1.33) | 1.04 (0.62-1.75) |
| Muslim x Ramadan during the third trimester | 0.97 (0.70-1.33) | 1.13 (0.65-1.98) |
| Muslim x Born during Ramadan | 1.10 (0.76-1.60) | 1.01 (0.51-1.97) |
| Ramadan exposure |  |  |
| Not exposed | Ref. | Ref. |
| Probably not exposed | 1.14 (0.84-1.56) | 0.67 (0.34-1.31) |
| Conceived during Ramadan | 1.16 (0.88-1.53) | 0.84 (0.49-1.45) |
| Ramadan during the first trimester | 1.16 (0.92-1.44) | 1.13 (0.75-1.71) |
| Ramadan during the second trimester | 1.08 (0.86-1.34) | 1.06 (0.70-1.59) |
| Ramadan during the third trimester | 1.04 (0.81-1.32) | 1.12 (0.71-1.74) |
| Born during Ramadan | 1.01 (0.75-1.34) | 1.01 (0.59-1.72) |
| Muslim |  |  |
| Non-Muslim | Ref. | Ref. |
| Muslim | 0.88 (0.69-1.13) | 1.06 (0.68-1.65) |
| Age | 1.30 (1.26-1.34) | 1.40 (1.32-1.48) |
| Age^2^ | 1.00 (1.00-1.00) | 1.00 (1.00-1.00) |
| Birth months |  |  |
| January | Ref. | Ref. |
| February | 1.02 (0.84-1.24) | 1.00 (0.71-1.42) |
| March | 1.00 (0.83-1.21) | 1.16 (0.84-1.61) |
| April | 1.00 (0.82-1.20) | 1.22 (0.88-1.68) |
| May | 0.94 (0.77-1.13) | 1.11 (0.80-1.54) |
| June | 1.03 (0.85-1.24) | 0.97 (0.69-1.36) |
| July | 0.99 (0.83-1.20) | 1.22 (0.89-1.67) |
| August | 0.91 (0.75-1.10) | 0.87 (0.62-1.24) |
| September | 0.93 (0.77-1.13) | 1.05 (0.75-1.46) |
| October | 0.93 (0.77-1.12) | 0.98 (0.70-1.38) |
| November | 0.96 (0.80-1.16) | 0.98 (0.70-1.37) |
| December | 0.98 (0.81-1.19) | 1.12 (0.81-1.56) |
| Constant | <0.01 (<0.01-<0.01) | <0.01 (<0.01-<0.01) |
| Observations | 20,575 | 20,575 |
| Pseudo R^2^ | 0.07 | 0.04 |

**Table A2**. Robustness checks for the association between in utero Ramadan exposure and mean arterial pressure in adult offspring (≥50 years old, n=14,194), by sex (only interaction terms are shown)

| **Variables** | **Female** | | **Male** | |
| --- | --- | --- | --- | --- |
|  | **Coef.** | **p-value** | **Coef.** | **p-value** |
| Model 1* (adj. R^2^$=$0.03 and 0.02) |  |  |  |  |
| Muslim x Not exposed (group (i) + (ii)) | Ref. | Ref. | Ref. | Ref. |
| Muslim x In utero Ramadan (group (iii) to (vii)) | -1.15 | 0.16 | 0.29 | 0.73 |
| Model 2^†^ (not exposed vs. exposed/probably not exposed) (adj. R^2^$=$0.03 and 0.03) | | | | |
| Muslim x Certainly not exposed (group (i)) | Ref. | Ref. | Ref. | Ref. |
| Muslim x Probably not exposed (group (ii)) | 1.44 | 0.36 | -2.06 | 0.22 |
| Muslim x In utero Ramadan (group (iii) to (vii) | -0.65 | 0.51 | -0.42 | 0.68 |
| Model 3^‡^ (adj. R^2^$=$0.03 and 0.03) |  |  |  |  |
| Muslim x Certainly not exposed (group (i)) | Ref. | Ref. | Ref. | Ref. |
| Muslim x Probably not exposed (group (ii)) | 1.43 | 0.36 | -2.03 | 0.23 |
| Muslim x Conceived during Ramadan (group (iii)) | -0.86 | 0.53 | -0.23 | 0.88 |
| Muslim x Ramadan during the first trimester (group (iv)) | -0.59 | 0.60 | -0.98 | 0.40 |
| Muslim x Ramadan during the second trimester (group (v)) | -0.71 | 0.51 | -0.04 | 0.98 |
| Muslim x Ramadan during the third trimester (group (vi)) | -0.89 | 0.45 | -0.96 | 0.44 |
| Muslim x Born during Ramadan (group (vii)) | 0.08 | 0.96 | 1.00 | 0.51 |

Note:

* Adjusted for age, age^2^, birth months (categorical), Muslim, not exposed, exposed.

^†^ Adjusted for age, age^2^, birth months (categorical), Muslim, probably not exposed, exposed.

^‡^ Adjusted for age, age^2^, birth months (categorical), Muslim, probably not exposed, conceived during Ramadan, Ramadan during the first trimester, Ramadan during the second trimester, Ramadan during the third trimester, and born during Ramadan.

**Table A3**. Robustness checks for the association between in utero Ramadan exposure and random blood glucose in adult offspring (≥50 years old, n=14,192), by sex (only interaction terms are shown)

| **Variables** | **Female** | | **Male** | |
| --- | --- | --- | --- | --- |
|  | **Coef.** | **p-value** | **Coef.** | **p-value** |
| Model 1* (adj. R^2^$=$0.01 and <0.01) |  |  |  |  |
| Muslim x Not exposed (group (i) + (ii)) | Ref. | Ref. | Ref. | Ref. |
| Muslim x In utero Ramadan (group (iii) to (vii)) | -0.17 | 0.52 | -0.16 | 0.54 |
| Model 2^†^ (not exposed vs. exposed/probably not exposed) (adj. R^2^$=$0.01 and <0.01) | | | | |
| Muslim x Certainly not exposed (group (i)) | Ref. | Ref. | Ref. | Ref. |
| Muslim x Probably not exposed (group (ii)) | -0.06 | 0.91 | -0.07 | 0.90 |
| Muslim x In utero Ramadan (group (iii) to (vii) | -0.19 | 0.55 | -0.18 | 0.57 |
| Model 3^‡^ (adj. R^2^$=$0.01 and <0.01) |  |  |  |  |
| Muslim x Certainly not exposed (group (i)) | Ref. | Ref. | Ref. | Ref. |
| Muslim x Probably not exposed (group (ii)) | -0.06 | 0.91 | -0.07 | 0.90 |
| Muslim x Conceived during Ramadan (group (iii)) | -0.15 | 0.73 | 0.13 | 0.77 |
| Muslim x Ramadan during the first trimester (group (iv)) | -0.14 | 0.69 | -0.18 | 0.63 |
| Muslim x Ramadan during the second trimester (group (v)) | -0.25 | 0.48 | -0.20 | 0.57 |
| Muslim x Ramadan during the third trimester (group (vi)) | -0.25 | 0.51 | -0.15 | 0.70 |
| Muslim x Born during Ramadan (group (vii)) | -0.08 | 0.85 | -0.54 | 0.25 |

Note:

* Adjusted for age, age^2^, birth months (categorical), Muslim, not exposed, exposed.

^†^ Adjusted for age, age^2^, birth months (categorical), Muslim, probably not exposed, exposed.

^‡^ Adjusted for age, age^2^, birth months (categorical), Muslim, probably not exposed, conceived during Ramadan, Ramadan during the first trimester, Ramadan during the second trimester, Ramadan during the third trimester, and born during Ramadan.
